# Closing the global cancer divide- performance of breast cancer care services in a middle income developing country

**DOI:** 10.1186/1471-2407-14-212

**Published:** 2014-03-20

**Authors:** Gerard CC Lim, Emran N Aina, Soon K Cheah, Fuad Ismail, Gwo F Ho, Lye M Tho, Cheng H Yip, Nur A Taib, Kwang J Chong, Jayendran Dharmaratnam, Matin M Abdullah, Ahmad K Mohamed, Kean F Ho, Kananathan Ratnavelu, Chiao M Lim, Kin W Leong, Ibrahim A Wahid, Teck O Lim

**Affiliations:** 1 Kuala Lumpur Hospital, Kuala Lumpur, Malaysia; 2 Universiti Malaya Medical Centre, Kuala Lumpur, Malaysia; 3 Mahkota Cancer Centre, Melaka, Malaysia; 4 Sime Darby Medical Centre, Petaling Jaya, Malaysia; 5 Mount Miriam Cancer Hospital, Pulau Pinang, Malaysia; 6 Nilai Medical Centre, Nilai, Malaysia; 7 Gleneagles Medical Centre Pinang, Pinang, Malaysia; 8 Beacon International Specialist Centre, Petaling Jaya, Malaysia; 9 ClinResearch SB, Kuala Lumpur, Malaysia

**Keywords:** Breast cancer, Cancer burden, Developing country, Performance measurement, Healthcare quality, Health policy, Health services research, Health system research

## Abstract

**Background:**

Cancer is the leading cause of deaths in the world. A widening disparity in cancer burden has emerged between high income and low-middle income countries. Closing this cancer divide is an ethical imperative but there is a dearth of data on cancer services from developing countries.

**Methods:**

This was a multi-center, retrospective observational cohort study which enrolled women with breast cancer (BC) attending 8 participating cancer centers in Malaysia in 2011. All patients were followed up for 12 months from diagnosis to determine their access to therapies. We assess care performance using measures developed by Quality Oncology Practice Initiative, American Society of Clinical Oncology/National Comprehensive Cancer Network, American College of Surgeons’ National Accreditation Program for Breast Centers as well as our local guideline.

**Results:**

Seven hundred and fifty seven patients were included in the study; they represent about 20% of incident BC in Malaysia. Performance results were mixed. Late presentation was 40%. Access to diagnostic and breast surgery services were timely; the interval from presentation to tissue diagnosis was short (median = 9 days), and all who needed surgery could receive it with only a short wait (median = 11 days). Performance of radiation, chemo and hormonal therapy services showed that about 75 to 80% of patients could access these treatments timely, and those who could not were because they sought alternative treatment or they refused treatment. Access to Trastuzumab was limited to only 19% of eligible patients.

**Conclusions:**

These performance results are probably acceptable for a middle income country though far below the 95% or higher adherence rates routinely reported by centres in developed countries. High cost trastuzumab was inaccessible to this population without public funding support.

## Background

Cancer is the leading cause of deaths and disability in the world, and a widening disparity in cancer burden has emerged between high income and low-middle income countries (LMIC) [1,2]. Once thought to be exclusively a burden for developed countries, developing countries today bear an increasing proportion of the burden; In 1970, 15% of newly reported cancers were in developing countries, compared with 56% in 2008 [3]. By 2030, the proportion is expected to be 70% [3-5]. This is due to population growth, ageing and changing lifestyles (smoking, diet etc.). Developing countries also bear an increasing share of the burden in cancer deaths; two-thirds of the 7 · 6 million deaths every year from cancer worldwide occur in LMIC [4,5]. This is due to improving survival in developed countries in the past 3 decades as a result of earlier detection and new and more effective treatments [6], but little of these advances are accessible to most people in LMIC.

Closing this cancer divide between rich and poor countries is not just an ethical imperative. There is sound economic justification too for reducing avoidable cancer deaths which are costly in economic productivity terms. The Global Task Force on Expanded Access to Cancer Care and Control in Developing Countries has recently presented a compelling case for comprehensive action on expanded access to cancer care and control [1,2]. In responding to its call for action, we had reviewed the cancer care services in Malaysia, a middle income country, and it was immediately obvious there was hardly any data at all to inform national cancer care planning. Similarly there is little granularity to the performance of cancer services in other developing countries too.

Evaluating the performance of cancer services however is challenging. Unlike for HIV, with which cancer care is often compared [7], where access could simply be measured by the number of HIV patients treated with anti-retroviral drugs, there is no simple method for measuring access to cancer care. Cancer care is far more complex and multi-faceted. Early diagnosis is important, which requires an active screening service and tissue diagnosis; tumor characteristics are heterogeneous and there are multiple treatment modalities which have to be individually tailored guided by specific tumor characteristics while taking into consideration patient’s preference and local availability of treatments. Cancer is increasingly a chronic disease and its care could stretch over years, which further complicates its measurement.

Fortunately, there have been recent advances in the development of evidence based rigorous and scientifically sound performance metrics [8-12]. These measures basically recommend a specific treatment modality for a sub-group of cancer patients defined by specific tumor characteristics, and further specify a time interval from diagnosis when treatment should be initiated. These measures have been adopted by national bodies tasked with healthcare quality oversight such as the National Quality Forum in US [13]. This has helped standardize the collection of cancer care data and enable the evaluation of the extent to which cancer care in a country adhere with current evidence as described by the performance measures. It has also helped identify factors contributing to sub-optimal care, so that appropriate strategies and interventions could be implemented to improve the delivery of services.

We adopted these performance metrics to measure performance of cancer care services in Malaysia, a middle income developing country in South East Asia with a population of 28.9 million and GDP per capita of RM 30,420 (~USD9,000) in 2011 [14]. In 2012, Malaysia’s age standardized cancer incidence was 143.6 per 100,000 population, cancer mortality 85.8 per 100,000 population, giving a Mortality: Incidence ratio of 60% [15]. Historically, the Malaysian health care system, like other former British colonies such as Hong Kong and Sri Lanka in the region, has retained a tax-funded public health service, much like the British National Health Service, alongside a private sector in a mixed health economy. However, as the economy matures, the private health sector, largely financed by private insurance, employer provided benefits or out-of-pocket payment has become increasingly sizeable. In 2006, annual health spending was 4.3% of GDP, of which public and private finance accounted for 45% and 55% respectively [16]. Finance and provision for cancer care in Malaysia follow the same public-private split; the public sector where patients receive publicly funded therapy and the private sector where patients pay out of pocket or their health insurance or employers fund treatment.

## Methods

We conducted a multi-center, retrospective observational cohort study to measure the performance of breast cancer care services in Malaysia. A central ethics committee, the Ministry of Health’s Medical and Research Ethics Committee, has approved the study and granted waiver from the requirement to obtain individual informed consent from patients. The waiver is justified by this being an observational study based entirely on data abstracted from medical record, and that such data are already routinely collected for healthcare quality assurance purpose.

### Study population

The study population consisted of women with breast cancer diagnosed in year 2011. The 9 largest cancer centres (centers with 2 or more mega-voltage machines) from both the public and private sector in Peninsular Malaysia were invited to participate of which 8 agreed to. Each centre was required to enroll all patients diagnosed and treated in the year 2011. Only Malaysian patients with primary breast carcinoma are included. Cases are identified through hospital register as well as operative surgery, chemotherapy and radiotherapy records. Case ascertainment for 3 of the centers was independently verified to be complete (100%).

### Study assessment and definitions

At enrollment, data were abstracted from patients’ medical and histo-pathology (HPE) reports by trained data collectors. Demographic data abstracted include age, sex race and nationality; tumor characteristics include histologic type, grade, location, extent, and size; lymph node and distant organ metastases. Staging of disease was based on the American Joint Committee on Cancer (AJCC 7^th^ Edition) criteria. AJCC stage I or II disease were considered early breast cancer (EBC), stage III locally advanced BC (LABC) and stage IV metastatic BC (MBC). After enrollment, all patients were followed up for 12 months to collect data on their subsequent exposure to cancer-directed therapies, which were abstracted from medical, operative surgery, chemotherapy and radiotherapy records.

For the purpose of measuring breast cancer care performance, we adopted the performance measures (Table 1) developed by Quality Oncology Practice Initiative (QOPI) [8,9], American Society of Clinical Oncology/National Comprehensive Cancer Network (ASCO-NCCN) [10,11], American College of Surgeons’ National Accreditation Program for Breast Centers (NAPBC) [12] as well as our local clinical practice guideline [17].

**Table 1:** Performance measures for evaluating breast cancer care services in Malaysia

**#**	**Performance measure**	**Source**
	*Diagnostic services*	
	Pathology report confirming malignancy	QOPI [8,9]
	Biomarker information	QOPI [8,9]
	*Treatment services*	
	Surgery for women under age 70 with Stage I to III breast cancer within 60 days of date of diagnosis	Malaysian guideline [17]
	Adjuvant multi-agent (combination) chemotherapy for women under age 70 with Stage I (T1c) to III ER/PR negative breast cancer within 120 days of date of diagnosis	ASCO-NCCN [10,11]
	Radiation therapy for women under age 70 with Stage I to III breast cancer who had breast conserving surgery for breast cancer within 1 year (365 days) of date of diagnosis	ASCO-NCCN [10,11]
	Radiation therapy for women under age 70 who had mastectomy for breast cancer with node + (four or more positive regional lymph nodes) within 1 year (365 days) of date of diagnosis	NAPBC [12]
	Tamoxifen or Aromatase Inhibitor for women greater than age 17 with Stage I (T1c) to III ER or PR positive breast cancer within 1 year (365 days) of date of diagnosis	ASCO-NCCN [10,11]
	Trastuzumab therapy for women greater than age 17 with Stage I (T1c) to III HER2 positive breast cancer within 1 year (365 days) of date of diagnosis	QOPI [8,9]

### Independent data audit

A copy of the HPE report was retrieved for all patients enrolled from all sites to verify tumor diagnosis and characteristics. In addition, patients’ demographic and treatment data from 3 sites were also subjected to independent data verification against source documents on site. The accuracy of the collected data with respect to demographics, surgery, radiotherapy, chemotherapy, hormonal therapy and trastuzumab treatment were all >95%.

### Statistical methods

Continuous variables are described by summary statistics such as mean, median, and standard deviation and categorical (nominal/ordinal) variables by the frequencies of each category. The precision of the estimates is described by 95% confidence interval (CI).

## Results

The 8 participating centres enrolled a total of 889 patients in 2011. One hundred and thirty two patients were excluded because of incomplete data (121 patients for date of diagnosis, 11 for tumor staging). Thus the final sample size was 757 subjects, which represent about 20% of all incident breast cancers in Malaysia in 2011.

### Patients’ demographic and tumor characteristics

Table 2 shows the patients’ demographic and tumor characteristics. The mean age of the women was only 53 years; about 40% was aged <50 years. 61% of patients were diagnosed with Early Breast Cancer (Stage 1 or 2, EBC), another 27% with Locally Advanced Cancer and 11% with late stage metastatic cancer. 65% were ER+, 57% PR+, 28% HER2+ and 12% triple negative.

**Table 2:** Patient and tumor characteristics at diagnosis

**Patient characteristics**	**Statistics**	**Results**
Number of patients	Number	757(100%)
Age, years	Mean (SD)	53(11)
	Median (IQR)	53(46, 61)
	(Min, Max)	(23, 87)
Age distribution	No. (%) age < 40	88(12)
	No. (%) age 40 to 49	199(26)
	No. (%) age 50 to 59	262(35)
	No. (%) age > =60	208(27)
Sex	No. (%) male	0(0)
	No. (%) female	757(100)
Race	No. (%) Malay	264(35)
	No. (%) Chinese	377(50)
	No. (%) Indian	95(13)
	No. (%) Orang Asli	1(0)
	No. (%) Bumiputera Sabah	0(0)
	No. (%) others	20(3)
Stage at diagnosis*	No. (%) Early Breast Cancer (EBC)	463(61)
	No. (%) Locally Advanced Breast Cancer (LABC)	207(27)
	No. (%) Metastatic Breast Cancer (MBC)	87(11)
Tumor size*	No (%) T1 (1 to 20 mm)	207(27)
	No (%) T2 (21 to 50 mm)	246(32)
	No (%) T3 (> 50 mm)	80(11)
	No (%) unknown	224(30)
Regional node*	No (%) negative node	206(27)
	No (%) 1–3 positive node	117(15)
	No (%) 4–10 positive node	75(10)
	No (%) >10 positive node	49(6)
	No (%) unknown	310(41)
Tumor histology*	No (%) infiltrating duct carcinoma, NOS	653(86)
	No (%) intraductal carcinoma, non-infiltrating, NOS	25(3)
	No (%) other carcinomas	79(10)
Grading*	No (%) grade 1	50(7)
	No (%) grade 2	261(34)
	No (%) grade 3	256(34)
	No (%) no information	190(25)
Biomarkers	No. (%) ER+	494(65)
	No. (%) missing information on ER	37(5)
	No. (%) PR+	434(57)
	No. (%) missing information on PR	40(5)
	No. (%) ER+/PR+	516(68)
	No. (%) missing information on ER & PR	36(5)
	No. (%) HER2 ISH + or IHC + if ISH missing or unknown	209(28)
	No. (%) missing information on HER2	169(22)
	No. (%) HER2 -	379(50)
	No. (%) Triple positive (ER+, PR + HER+)	95(13)
	No. (%) Triple negative (ER-, PR- HER-)	94(12)
	No. (%) missing information ER, PR and/or HER	173(23)

*Results on Staging and Histologic findings (tumor size, node, histology grade) may not be consistent with one another because data on the former were abstracted from patients’ medical records or treatment plan while latter were abstracted from histo-pathology report submitted by participating sites.

### Cancer care performance

Table 3 summarizes the performance results of Malaysian cancer diagnostic services. For patients first presenting at a treatment centre, it took a median of 9 days to arrive at a diagnosis of cancer. All patients (100%) had a pathology report confirming malignancy. One hundred and seventy three patients however had no information on one or more tumor biomarkers (5% ER, 5% PR and 22% HER2).

**Table 3:** Performance of cancer diagnostic services for a breast cancer cohort in Malaysia, in year 2011

**#**	**Performance of cancer diagnostic services**	**N = 757**
1.	Median (IQR) duration from first presentation at site to diagnosis, days	9(4, 9)
2.	Number (%) of patients with Pathology report confirming malignancy	757(100)
3.	Number (%) of patients with information on ER	720(95)
4.	Number (%) of patients with information on PR	717(95)
5.	Number (%) of patients with information on HER2	588(78)
6.	Number (%) of patients without information on ER or PR or HER2	173(23)

Table 4 summarizes the performance results of Malaysian cancer treatment services. Breast cancer surgery was highly accessible; 671 (89%) patients had surgery with a median time from diagnosis to surgery of only 11 days. Only 25% of patients underwent breast conserving surgery.

**Table 4:** Performance of cancer treatment services for a breast cancer cohort in Malaysia in year 2011

**#**	**Performance measures for cancer treatment services**	**Number of patients eligible for inclusion for the performance measure (Denominator)**	**Percent of patients whose care adhere with performance measure**	**95% CI of percent of patients whose care adhere with performance measure**
1.	Patients under age 70 with Stage I to III Breast cancer who received Surgery within 2 months of diagnosis	610	82%	(79, 85)
2.	Patients under age 70 with Stage I (T1c) to III ER/PR negative Breast cancer who received Chemotherapy within 4 months of diagnosis	171	75%	(68, 81)
3.	Patients under age 70 with Stage I to III Breast cancer who received Radiation therapy after breast conserving surgery within 1 year of diagnosis	159	77%	(69, 83)
4.	Patients under age 70 Node + Breast cancer who received Radiation therapy after mastectomy within 1 year of diagnosis	89	81%	(71, 88)
5.	Patients under age 70 with Stage I (T1c) to III ER or PR positive Breast cancer who received Tamoxifen or AI within 1 year of diagnosis	428	76%	(72, 80)
6.	Patients under age 70 with Stage I (T1c) to III HER2 positive Breast cancer who received Trastuzumab within 1 year of diagnosis	172	19%	(14, 26)

Performance for the 3 treatment modalities, radiation, chemotherapy and hormonal therapy, were comparable. Four hundred and seventy three (62%) patients had chemotherapy with a median time from diagnosis to treatment of 51 days. Most had an alkylating agents (95%), anthracycline antibiotics (86%) or anti-metabolites (76%); only 41% of patients had a taxane. 75% of patients eligible for chemotherapy had care that adhere with the performance measure and received therapies within the prescribed time.

Four hundred and sixty nine (62%) patients had radiotherapy with a median time from diagnosis to treatment of 194 days. Half of them had whole breast external irradiation while 38% had tumour bed (boost) irradiation. 77% of eligible patients had radiotherapy after breast conserving surgery within the prescribed time, while 81% who had radiotherapy after mastectomy had care that adhered with the performance measure. Four hundred and thirty (57%) patients had hormonal therapy with a median time from diagnosis to treatment of 171 days. Most had tamoxifen (85%), only 13% of patients had an aromatase inhibitor. And 76% of eligible patients had care that adhered with the performance measure. For a subset of these patients (N = 89) whose care did not adhere with the performance measures, further investigations showed the common reasons for non- adherence were the patients having sought care in another centre (33%), sought alternative or traditional treatment (16%) or they had refused treatment (50%).

For patients with HER2 positive cancer, access to targeted therapy (trastuzumab) was very limited; only 19% of eligible patients could be treated.

## Discussion and conclusion

Breast cancer is a common disease across the world but outcomes vary significantly between high and low income countries. Most women diagnosed with breast cancer in high-income countries can reasonably expect to be cured and enjoy a long life expectancy. Such progress has been made possible by screening programmes that enable early detection and by the use of multiple modality treatments. However, in low and middle income countries, under-resourced and under-performing health services continue to fail to deliver adequate screening and treatments leading to poor outcomes for patients with breast cancer.

In the year 2011, we measure the performance of breast cancer care to inform our advocacy for better cancer services in Malaysia. The study sample is large (20% of incident cases in 2011) but it is not likely to be representative of the population it aims to describe. The cancer care performance results presented here are likely to be better than they really were (optimistic bias). Firstly, patients were enrolled from 8 of the 9 leading cancer centers in Peninsular Malaysia where cancer specialist manpower and physical infrastructure are concentrated. Clearly large number of BC patients in Malaysia received care in less well-resourced settings and they are not included in this study. Secondly, only patients with complete data can be included in the performance measure analysis. For example, for the measure “Patients under age 70 with Stage I to III ER/PR negative Breast cancer who received Chemotherapy within 4 months of diagnosis”, to be included in this analysis require a patient to have complete data (non-missing) on date of diagnosis and date treatment was started, details on staging (T1c or Stage II or III), ER and PR, age (18–69 years) and treatment course (only the first is counted). However as shown in Table 2, critical data to inform clinical decision making were frequently missing (30% for tumor size, 22% for HER2 etc.) in the real world practice in developing countries. In so far that patients with more complete information are likely to receive better care, the results are optimistically biased. Thus, the cancer care performance results presented here represent the upper bound of what is achievable in a middle income country. The results however are unlikely to be affected by selection bias due to selective enrolment as we had 100% case ascertainment within centres. Similarly selective reporting is also unlikely as an independent audit has verified the accuracy of the data. The estimate of non-adherence with performance measure does not take into account individual physician practice style or patient preference; the performance measures were designed to ignore such considerations [10].

The performance results were mixed. Late presentation was 40% which is the same as more than 10 years ago when this was first reported [18,19], indicating little progress at all in cancer screening services in the past 10 years, notwithstanding stage shift due to changes in AJCC definition over the years. On the positive side, we found timely access to diagnostic and breast surgery services. The interval from presentation to tissue diagnosis was short, and all who needed surgery could receive it with only a short wait. Performance of radiation, chemotherapy and hormonal therapy services were probably acceptable. About 75 to 80% of patients could access these treatments in a timely fashion, and those who could not were because they sought alternative treatment or treatment elsewhere, or they simply refused treatment. These performance results are probably creditable for a middle income country though obviously they are far below the 95% or higher adherence rates routinely reported by many centres of excellence in developed countries [20-23]. Access to trastuzumab was the only problematic area in Malaysian cancer care. This was entirely due to the high cost and inadequate public funding for the treatment.

The results presented here merely describe the mean performance of the cancer care provided by 8 leading centers in Malaysia, 2 of these centres are publicly owned. We did not address the likely variation in cancer care performance between centres or between public and privately owned centres for several reasons. The study protocol explicitly prohibits comparative performance analysis between centres. This was necessary to attract centers to voluntarily contribute data to this study mostly at their own expense. Besides the sensitivity of comparative performance analysis, defining whether a patient is private or public is not straightforward in a highly fragmented cancer care system, such as the one in Malaysia. The boundary between centers are ill-defined and porous. One of the public centre in our sample also treat fees paying patients within the same centre alongside public patients. Another public hospital in the sample routinely outsource radiotherapy services to private centres. Oncologists in public hospitals commonly (probably almost all of them) practice privately and many of their private patients see them in their public practice too and vice versa. And finally in the course of their cancer care, all patients frequently move between centres whether within the public or private sector or between the 2 sectors.

A high performing health service is crucial to translating medical advances into improved health for the population. To our knowledge, this is the first time cancer service in a developing country has been subjected to measurement using standardized performance metrics. Clearly there is room for improvement. The results are useful too as a baseline against which future improvement will be measured. The results also highlight the importance of routine performance measurement in healthcare, which is under-developed in many developing countries despite their high cancer burden [24]. Investment in health without monitoring the return on the investment and without holding the recipients of health funding and providers of healthcare accountable would be unconscionable [25].

Many strategies and solutions have been proposed to improve cancer services in developing countries [1,26,27]. First, the fragmented cancer services typically found in many middle-income countries including Malaysia need to be reformed; we should explore novel models of care delivery [28,29]. Second, the innovative financing, pricing and procurement strategies which had successfully aided the fight against communicable diseases such as HIV/AIDS, could similarly be employed in cancer care to improve access to high cost medicines. The pharmaceutical industry has been responsive to the needs of developing countries by offering “access schemes” [7] or second brand product [30] which substantially reduce prices and render the treatment more accessible. Ultimately, leadership and advocacy for cancer care needs to be strengthened. Cancer service in Malaysia as described in this study has much to learn yet from other high cost services such as dialysis. Able leadership and the will to radically reform the financing and delivery of dialysis service informed by rigorous health policy research were crucial to achieving universal access to dialysis in Malaysia [31]. We need to do the same for cancer care.

## Competing interests

The authors declare no competing interests, whether of the financial or non-financial kind.

## Authors’ contributions

GCCL, ENA, GFH, CHY, NAT, KJC, JD, MMA, AKM, KFH, KR, CML, KWL, IAW and TOL jointly conceived the study idea, wrote the report and provided subject matter expertise. TOL in addition, designed the study, manage the project and data and conducted the data analysis. All authors read and approved the final manuscript.

## Pre-publication history

The pre-publication history for this paper can be accessed here:

http://www.biomedcentral.com/1471-2407/14/212/prepub
